# Mirror of Motherhood: Appearance Schema, Appearance Anxiety, and Body Image Quality of Life in Women

**DOI:** 10.7759/cureus.105574

**Published:** 2026-03-20

**Authors:** Gayathri R, Paramita Datta

**Affiliations:** 1 Applied Psychology, Faculty of Behavioural and Social Sciences (FBSS), Sri Ramachandra Institute of Higher Education and Research (Deemed to be University), Chennai, IND

**Keywords:** appearance anxiety, appearance schema, body image quality of life, childcare, interpersonal relationships, maternal well-being, postpartum body image

## Abstract

The postpartum period is marked by profound physical and psychosocial changes that can significantly influence how women perceive their bodies and overall emotional well-being. Despite growing recognition of postpartum body dissatisfaction, limited research has explored the cognitive factors that may contribute to these concerns. This study aimed to examine the relationship between appearance schema, appearance anxiety, and body image-related quality of life among postpartum women. A cross-sectional study was conducted among 92 postpartum women (aged 20-40 years) in Tamil Nadu, India. Participants completed standardized measures, including the Appearance Schema Inventory-Revised (ASI-R), Appearance Anxiety Inventory (AAI), and Body Image Quality of Life Inventory (BIQLI). Pearson's correlation analyses were used to assess associations between variables. Greater cognitive investment in appearance was significantly associated with higher levels of appearance anxiety (r = .338, p < .001) and poorer body image quality of life (r = −.272, p = .009). Appearance anxiety and its subcomponents-threat monitoring, camouflaging, and avoidance-were also negatively associated with quality of life (p < .05). In particular, women who closely linked their self-worth to appearance reported lower overall life satisfaction related to body image. The findings suggest that how women think about and value their appearance plays an important role in shaping emotional distress and quality of life during the postpartum period. Incorporating early psychological screening and cognitive-based support into postpartum care may help promote healthier body image and improved maternal well-being.

## Introduction

The transition from womanhood to motherhood is a profound and multifaceted journey, marked by both joy and immense challenges. Women undergo significant physical, psychological, and social transformations postpartum, which can deeply influence their mental well-being. The postpartum period is often accompanied by societal pressures to “bounce back” and regain a pre-pregnancy physique, an unrealistic expectation that can contribute to body dissatisfaction and self-esteem issues [1]. These expectations are reinforced by cultural norms that disproportionately emphasize women’s appearance over their capabilities and achievements [2]. Study shows this unrealistic idea of achieving a toned and slender postpartum body is strongly emphasized by society [3]. Empirical research indicates that postpartum women experience heightened body dissatisfaction within the initial weeks after childbirth compared to pre-pregnancy periods [4]. The internalization of body image ideals, coupled with feelings of shame and avoidance, can lead to adverse outcomes, including disordered eating, increased body mass index (BMI), reluctance to breastfeed, and poor mental health [5]. In contrast, a positive body image fosters self-acceptance and strengthens maternal-infant bonding [3].

While existing literature acknowledges the co-existence of body dissatisfaction and acceptance in postpartum women, research predominantly focuses on physiological changes, neglecting the psychological aspects that shape postpartum body image [6]. Most of the studies have a unidimensional focus, which neglects psychological aspects of this change and rigidly focuses on the physiological aspect. There seems to be a well-established tradition in postpartum care and obstetric as well as public health research to focus mainly on the antepartum body to minimize the risks of pregnancy and birth [7].

The cultural context significantly influences how women perceive their postpartum bodies. In many societies, including India, the postpartum period holds deep cultural and societal significance [8]. Because postpartum body changes are frequently unanticipated and unpredictable, body-related dissatisfaction might be significant. Society often places higher emphasis on appearance for women as they are evaluated by their physical appearance rather than their abilities and achievements [2]. Appearance schema refers to deeply ingrained cognitive frameworks that influence how individuals perceive, evaluate, and respond to their physical appearance. Appearance schemas, shaped by cultural frameworks, play a crucial role in determining body image satisfaction. These schemas consist of evaluative (self-ideal discrepancies, body satisfaction-disatisfaction) and investment components (self-schemas and behavioural approaches) [9]. The greater the discrepancy between a woman’s ideal and perceived body image, the higher the psychological distress experienced [10].

Anxiety is a common postpartum concern, with prevalence estimates ranging from 20% to 30% in community settings [11]. Postpartum anxiety is common and ranges from a healthy level of concern that supports optimal caregiving to severe conditions that may negatively impact the baby’s health [12]. Research indicates postpartum anxiety disorders (PPAD) are diagnosed in 4%-39% of pregnant women and 16% of women in the postpartum period [13]. Left unaddressed, postpartum anxiety can lead to feelings of inadequacy, guilt, and impaired caregiving, potentially resulting in long-term consequences for both mother and child [14].

Understanding postpartum body image and its implications is critical for promoting women’s well-being. The distress associated with body dissatisfaction extends beyond physical appearance, influencing social functioning, intimate relationships, and overall quality of life [15]. Body image quality of life refers to the extent to which an individual’s perception of their body influences their overall quality of life, encompassing physical, psychological, and social domains [16]. Body image concerns in postpartum women can profoundly affect their well-being, disrupting daily life, emotional stability, and interpersonal relationships. The postpartum period is often characterized by a heightened vulnerability to body dissatisfaction, which can lead to impaired self-esteem, social withdrawal, and relational conflicts [17].

In this above backdrop, the present study aims to examine how appearance schema and appearance anxiety are related and affect body-image quality of life in postpartum women.

## Materials and methods

Participants

At the beginning of the data collection process, 120 postpartum women attending a childcare center in Tamil Nadu, India, volunteered to take part in the study. A convenience sampling approach was used, and each interested participant was carefully screened according to the predetermined inclusion and exclusion criteria. Following this screening process, 92 women satisfied the eligibility requirements and completed all study measures. The remaining 28 women were not included in the final analysis because they did not meet the eligibility criteria, submitted incomplete responses, or chose to withdraw during data collection. Consequently, the final sample comprised 92 postpartum women whose responses were included in the analysis. Prior to data collection, a power analysis was conducted using G*Power (version 3.1.9.7, Heinrich-Heine-Universität Düsseldorf, Düsseldorf, Germany) to determine the appropriate sample size for examining bivariate correlations. Based on an anticipated medium effect size of 0.30, an alpha level of 0.05, and a desired statistical power of 0.80, the analysis indicated that at least 84 participants would be required. The final sample consisted of 92 women, thereby exceeding the minimum requirement and providing sufficient statistical power to reliably test the proposed relationships.

Women were eligible to participate if they were between 20 and 40 years of age and had given birth within the past twelve months. Both first-time mothers and those who had previously given birth were included, as long as they were within one year postpartum at the time of the study. Mothers of either single or multiple births were considered for participation. Women were not included if they had been previously diagnosed with a psychiatric condition, were experiencing significant postpartum medical complications, or were pregnant again during the period of data collection. These criteria were carefully defined to ensure that the study focused on the body image experiences of women undergoing a typical postpartum adjustment, without the added influence of severe medical or pre-existing psychological conditions.

Measures

Appearance Schema Inventory-Revised (ASI-R)

The Appearance Schema Inventory-Revised was used to measure the beliefs about appearance. It is a scientifically validated inventory, with 20 items, by Cash et al. (2004) [18]. The scale includes two components: self-evaluative salience and motivational salience. Self-evaluative salience refers to the extent to which individuals base their self-worth on their physical appearance. Motivational salience reflects how much attention individuals give to their appearance and the degree to which they engage in behaviors to manage or enhance it. The ASI-R includes reverse-scored items. It follows a 5-point Likert scale for responses (1 = Strongly Disagree, 2 = Mostly Disagree, 3 = Neutral, 4 = Mostly Agree, 5 = Strongly Agree).

Appearance Anxiety Inventory (AAI)

The Appearance Anxiety Inventory was used to measure the cognitive and behavioral aspects of body image-related anxiety. It is an empirically validated inventory, with 10 items, by Veale et al. (2014) [19]. The inventory consists of three components: threat monitoring, camouflaging, and avoidance. Threat monitoring refers to heightened awareness or vigilance toward perceived flaws in one’s appearance. Camouflaging involves efforts to conceal or disguise these perceived imperfections. Avoidance reflects the tendency to stay away from situations or activities due to concerns about appearance. Responses are recorded on a five-point Likert scale ranging from 0 (not at all) to 4 (all the time).

Body Image Quality of Life (BIQLI) Inventory

The Body Image Quality of Life Inventory was used to measure the quality of life of the postpartum women solely based on their body image [20]. It has 19 items and scores ranging from -3 to +3. 

Ethical Considerations

All procedures performed in this study involving human participants were in accordance with the ethical standards of the Institutional Research Ethics Committee, Sri Ramachandra Institute of Higher Education and Research (Deemed to be University). Ethical approval code for this study is CSP-III/24/DEC/13/484.

Procedure

To ensure linguistic and conceptual equivalence, all assessment tools were translated into the regional language using a standard translation process. Eligible participants were screened through a structured preliminary interview based on predefined inclusion criteria. Written informed consent was obtained from all participants prior to data collection. A total of 92 postpartum women were recruited using a convenience sampling technique. Data were collected in a controlled environment to ensure methodological rigor, procedural standardization and strict adherence to ethical guidance.

Statistical analysis was done by the Jamovi software (Version 2.6) (retrieved from https://www.jamovi.org).

**Figure 1:**
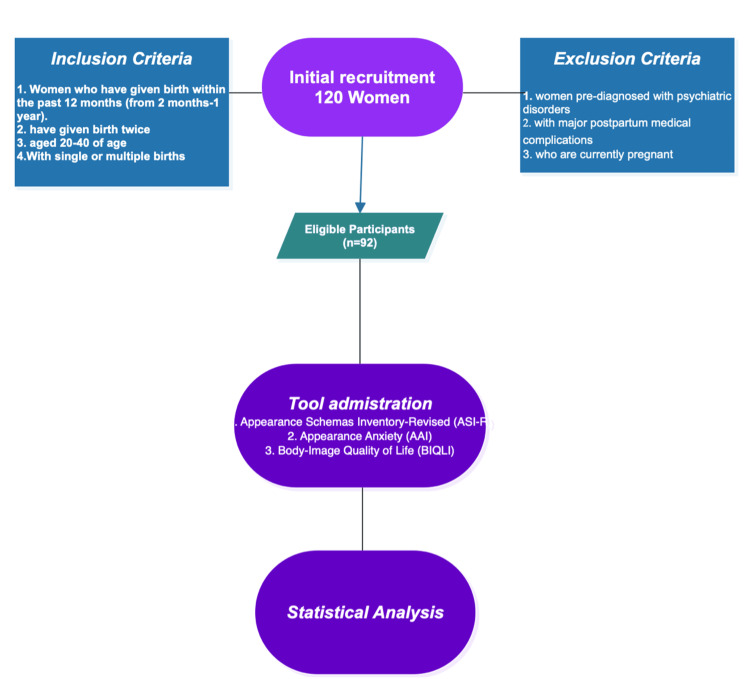
Overview of recruitment

Initially, 120 postpartum women expressed interest in participating in the study. All potential participants were screened based on the predefined inclusion and exclusion criteria. After this screening process, 92 women met the eligibility requirements and completed all study measures. The remaining 28 participants were excluded due to different reasons. The participant recruitment and screening process is illustrated in Figure 1.

## Results

Mean age of participants is 26 years, and standard deviation (SD) is 3.661. Table 1 presents the descriptive statistics for appearance schema, appearance anxiety, and body image quality of life. The average score for the appearance schema was 3.15, with a standard deviation of 0.59, indicating a moderate level of cognitive investment in appearance among the participants. The mean score for appearance anxiety was 16.93, with a standard deviation of 8.83, suggesting noticeable variability in anxiety related to appearance within the sample. For body image quality of life, the mean score was 0.33, with a standard deviation of 0.99, reflecting modest overall perceptions of how body image influenced participants’ daily functioning and well-being.

**Table 1:** Descriptive statistics of study variables

Variables	Mean	Standard deviation
Appearance schema	3.152	0.591
Appearance anxiety	16.93	8.834
Body image quality of life	0.326	0.995

Pearson’s correlation analysis indicated that higher levels of appearance schema were significantly associated with greater overall appearance anxiety (r = 0.338, p < .001). In addition, the appearance schema showed significant positive relationships with domains of appearance anxiety, including threat monitoring (r = 0.336, p < .001), camouflaging (r = 0.284, p = .006), and avoidance (r = 0.311, p = .003). These results suggest that women who place greater cognitive importance on their appearance are more likely to experience heightened anxiety and engage in appearance-related monitoring and coping behaviors. The detailed correlations are presented in Table 2.

**Table 2:** It shows the correlation between appearance schema and appearance anxiety * p <. 05, ** p < .01, *** p < .001

Variables	Appearance anxiety							
	Appearance anxiety total score		Threat monitoring		Camouflaging		Avoidance	
	Pearson’s correlation	P value	Pearson’s correlation	P value	Pearson’s correlation	P value	Pearson’s correlation	P value
Appearance schema total composite score	0.338***	< .001>	0.336**	< .001>	0.284**	0.006	0.311**	0.003
Self-evaluative salience	0.231*	0.027	0.231*	0.027	0.163	0.121	0.228*	0.029
Motivational salience	0.218*	0.037	0.229*	0.028	0.242*	0.020	0.146	0.164

Further analysis revealed that appearance schema was significantly and negatively associated with body image quality of life (r = −0.272, p = .009). This indicates that women who placed greater cognitive importance on their appearance tended to report lower satisfaction in life domains influenced by body image. When examining the subcomponents, self-evaluative salience showed a stronger negative relationship with body image quality of life (r = −0.306, p = .003). This suggests that women who closely tied their self-worth to their physical appearance experienced greater impairment in their overall quality of life. These findings are presented in Table 3.

**Table 3:** It shows the correlation between appearance schema and body image quality of life * p <. 05, ** p < .01, *** p < .001

Variables	Body image quality of life	
	Pearson’s correlation	P value
Appearance schema	-0.272**	0.009
Self-evaluative salience	-0.306**	0.003
Motivational salience	-0.032	0.764

Appearance anxiety was significantly and negatively associated with body image quality of life (r = −0.361, p < .001), indicating that higher appearance-related distress was linked to lower overall well-being. Among the subdomains, avoidance showed the strongest negative relationship (r = −0.392, p < .001), followed by threat monitoring (r = −0.347, p < .001) and camouflaging (r = −0.234, p = .025). These findings suggest that women who frequently worried about, monitored, or avoided situations due to appearance concerns tended to report poorer quality of life. The detailed results are presented in Table 4.

**Table 4:** shows the correlation between appearance anxiety and body image quality of life * p <. 05, ** p < .01, *** p < .001

Variables	Body image quality of life	
	Pearson’s correlation	P value
Appearance anxiety	-0.361***	< .001>
Threat monitoring	-0.347***	< .001>
Camouflaging	-0.234*	0.025
Avoidance	-0.392***	< .001>

## Discussion

This study investigated the relationships between appearance schema, appearance anxiety, and body image quality of life among women who gave birth in the past 12 months. The postpartum period is a critical transitional phase marked by significant physiological, psychological, and social changes that can profoundly impact self-perception and mental well-being. Even these difficulties persist beyond this period. Given that societal beauty standards often emphasize pre-pregnancy body ideals, many women experience heightened self-consciousness and distress regarding postpartum body-image changes. During the first year postpartum, women reported feeling heavier and physically weaker, along with a greater discrepancy between their current body shape and their ideal body image compared to their pre-pregnancy perceptions [21]. Depressive symptoms, greater physical comparison tendencies, and dieting tendencies also predicted body image of different types at 12 months post birth. The findings reveal that heightened cognitive investment in appearance and associated emotional distress significantly influence postpartum well-being. These results highlight the importance of understanding how postpartum women internalize appearance-related beliefs and how beliefs shape their emotional responses and behavioural patterns.

Consistent with previous findings [22], the appearance schema was positively associated with appearance anxiety, supporting the premise that individuals who strongly internalize appearance-related beliefs are more susceptible to psychological distress. This vulnerability may be particularly pronounced during the postpartum period, which is characterized by substantial physical, hormonal, and social transitions. The salience of sociocultural expectations during this phase may further intensify body-related concerns.

Cultural context may also play a moderating role in these relationships. In collectivistic societies such as India, the postpartum body is often situated within traditional norms and subject to heightened social scrutiny [8]. Such sociocultural pressures may amplify self-ideal discrepancies and contribute to increased emotional distress. Moreover, societal expectations that women should rapidly “return” to their pre-pregnancy appearance [2] may foster anxiety and insecurity, particularly in situations involving social comparison or perceived evaluation by others. 

In the present study, appearance schema demonstrated significant positive associations with the subdimensions of appearance anxiety (threat monitoring, camouflaging, and avoidance), providing empirical support for the proposed cognitive behavioral pathways. These findings suggest that postpartum women who place greater cognitive importance on appearance are more likely to engage in heightened vigilance toward perceived bodily flaws as well as behavioral strategies aimed at managing or concealing these concerns. Thus, the data indicate that appearance-related schemas not only contribute to emotional distress but also shape maladaptive coping responses during the postpartum period.

Self-evaluative salience and motivational salience of appearance schema demonstrated significant positive associations with appearance anxiety in the present study. Self-evaluative salience reflects the degree to which individuals anchor their self-worth to their physical appearance, whereas motivational salience denotes the importance attributed to maintaining or enhancing attractiveness [23]. These associations can be theoretically interpreted through the lens of self-discrepancy theory, which posits that psychological distress emerges when individuals perceive a discrepancy between their actual self and their internalized ideal or ought standards. During the postpartum period, rapid and often unpredictable bodily changes may intensify discrepancies between pre-pregnancy appearance ideals and current physical reality, thereby heightening vulnerability to appearance-related anxiety. Additionally, objectification theory provides a relevant lens for interpretation. Sociocultural emphasis on women’s bodies as objects of evaluation may foster self-objectification, wherein women internalize an observer’s perspective of their own bodies. In the postpartum context, heightened self-surveillance combined with limited capacity to invest time in appearance-related behaviors may exacerbate feelings of inadequacy and anxiety. Furthermore, role strain and identity transition theories suggest that the postpartum period involves a significant reorganization of identity.

Appearance anxiety showed a strong negative relationship with body image quality of life. This supports the notion that psychological distress rooted in body dissatisfaction can impair emotional well-being, social interaction, and maternal bonding [16]. Findings suggest that appearance schema and body image quality of life correlate, suggesting that high-schematic individuals have low quality of life. Negative self-beliefs regarding appearance can have adverse effects on psychosocial consequences like maladaptive eating patterns [15], depressive mood [24], social anxiety and inhibition [16], impaired sexual functioning [25], and poor self-esteem [26]. This suggests that individuals with high schema tend to have negative effects on various domains of life. In this case, postpartum women require a lot of care, reassurance, and support from family, partner, and society to live a quality life. Additionally, the self-evaluative salience correlated negatively with body image quality of life, which explains that postpartum women who place high expectations on their appearance tend to have lower quality of life, leading to detrimental effects on their health, childcare, and interpersonal relationships. Increased preoccupation with self leads to diminished quality of life in postpartum mothers, suggesting that restructuring cognitive beliefs can help gain positive self-perception and effective child care. Threat monitoring and body image quality of life show a negative correlation, emphasizing that more women monitor themselves, leading to diminished quality of life. Cash and Fleming hypothesized that poor body image quality of life is associated with dissatisfaction and preoccupation, more dysfunctional investment in oneself, stronger internalization of cultural beauty standards, and greater adiposity [16]. Furthermore, appearance schema, specifically self-evaluative salience, was negatively correlated with quality of life, suggesting that individuals who prioritize appearance may experience reduced satisfaction across life domains- childcare, intimacy, and interpersonal relationships. Prior studies have shown that negative body image can influence breastfeeding behaviour, maternal responsiveness, and infant attachment [27,28]. Also, reduced sexual satisfaction, influenced by perceived partner attitudes and cultural standards of beauty, has also been reported [29]

Collectively, the results underscore appearance schema as a cognitive vulnerability factor that influences emotional distress and quality of life in postpartum women.

Limitations

This homogeneous study population limits the generalizability of the findings to the wider population of postpartum women. Greater emphasis on distinguishing between primigravida and secundigravida mothers could have provided deeper insights into how previous childbirth experiences influence body image, appearance-related anxiety, and quality of life. A longitudinal design would offer a more comprehensive understanding of the evolving patterns of appearance schema, anxiety, and body image-related quality of life before, during, and after pregnancy.

Implications of this study and directions for future research

The findings of this study have several important implications for clinical practice, public health policy, and future research. Firstly, the strong associations between appearance schema, appearance anxiety, and diminished body image quality of life highlight the need for early psychological screening and support for women following childbirth. Integrating body image and self-perception assessments into routine postpartum care can help identify at-risk individuals and provide timely interventions.

Mental health professionals, particularly those working in maternal and child health settings, should be trained to recognize and address the psychological impact of appearance- related beliefs. Therapeutic approaches such as cognitive-behavioural therapy (CBT) may be beneficial in challenging maladaptive thought patterns and improving self-perception.

At a community and social level, awareness campaigns aimed at normalizing postpartum body changes and promoting body positivity can help shift cultural expectations and reduce the stigma surrounding maternal appearance. Educational initiatives can also engage partners and families to foster a more supportive and understanding environment for postpartum women. 

Moreover, given the influence of appearance-related distress on maternal functioning and infant bonding, addressing body image issues may have far-reaching effects on child development, maternal mental health, and family dynamics. The study provides a foundation for future longitudinal and interventional research that can explore the long-term effects of postpartum body image concerns and evaluate the effectiveness of body-positive interventions in diverse populations.

## Conclusions

This study demonstrates that postpartum women’s cognitive investment in appearance and associated anxiety significantly impact their quality of life. The findings clearly demonstrate that greater schematic investment in appearance and elevated appearance anxiety are linked to reduced body image satisfaction and impaired psychosocial functioning. These psychological challenges can affect multiple aspects of a new mother’s life, including emotional well-being, maternal-infant bonding, social engagement, and even physical health.

The results highlight the critical need for tailored interventions that challenge unrealistic appearance-related beliefs and encourage positive body image, especially in the vulnerable postpartum period.
